# Upregulation of Innate Antiviral Restricting Factor Expression in the Cord Blood and Decidual Tissue of HIV-Infected Mothers

**DOI:** 10.1371/journal.pone.0084917

**Published:** 2013-12-18

**Authors:** Nátalli Zanete Pereira, Elaine Cristina Cardoso, Luanda Mara da Silva Oliveira, Josenilson Feitosa de Lima, Anna Cláudia Calvielli Castelo Branco, Rosa Maria de Souza Aveiro Ruocco, Marcelo Zugaib, João Bosco de Oliveira Filho, Alberto José da Silva Duarte, Maria Notomi Sato

**Affiliations:** 1 Laboratory of Dermatology and Immunodeficiencies, LIM-56, Department of Dermatology, Medical School, Tropical Medicine Institute of São Paulo, University of São Paulo, São Paulo, São Paulo, Brazil; 2 Hospital das Clínicas, Department of Obstetrics and Gynecology, Medical School, University of São Paulo, São Paulo, São Paulo, Brazil; 3 Pediatrics Department, Medical Institute Prof. Fernando Figueira-IMIP, Recife, Pernambuco, Brazil; University British Columbia, Canada

## Abstract

Programs for the prevention of mother-to-child transmission of HIV have reduced the transmission rate of perinatal HIV infection and have thereby increased the number of HIV-exposed uninfected (HEU) infants. Natural immunity to HIV-1 infection in both mothers and newborns needs to be further explored. In this study, we compared the expression of antiviral restricting factors in HIV-infected pregnant mothers treated with antiretroviral therapy (ART) in pregnancy (n=23) and in cord blood (CB) (n=16), placental tissues (n=10-13) and colostrum (n=5-6) samples and compared them to expression in samples from uninfected (UN) pregnant mothers (n=21). Mononuclear cells (MNCs) were prepared from maternal and CB samples following deliveries by cesarean section. Maternal (decidua) and fetal (chorionic villus) placental tissues were obtained, and colostrum was collected 24 h after delivery. The mRNA and protein expression levels of antiviral factors were then evaluated. We observed a significant increase in the mRNA expression levels of antiviral factors in MNCs from HIV-infected mothers and CB, including the apolipoprotein B mRNA-editing enzyme 3G (A3G), A3F, tripartite motif family-5α (TRIM-5α), TRIM-22, myxovirus resistance protein A (MxA), stimulator of interferon (IFN) genes (STING) and IFN-β, compared with the levels detected in uninfected (UN) mother-CB pairs. Moreover, A3G transcript and protein levels and α-defensin transcript levels were decreased in the decidua of HIV-infected mothers. Decreased TRIM-5α protein levels in the villi and increased STING mRNA expression in both placental tissues were also observed in HIV-infected mothers compared with uninfected (UN) mothers. Additionally, colostrum cells from infected mothers showed increased tetherin and IFN-β mRNA levels and CXCL9 protein levels. The data presented here indicate that antiviral restricting factor expression can be induced *in utero* in HIV-infected mothers. Future studies are warranted to determine whether this upregulation of antiviral factors during the perinatal period has a protective effect against HIV-1 infection.

## Introduction

Effective measures for preventing infant HIV infection have reduced the transmission rate of perinatal HIV infection to approximately 2-5%; as a result the population of HIV-exposed uninfected (HEU) infants is growing [1,2]. These infants have a significantly increased risk of death during the first year of life and appear to suffer from weaknesses in their immune defenses [3,4]. However, the vertical transmission of HIV-1 is not an inevitable consequence of exposure; in the absence of treatment, 55-80% of infants exposed to HIV-1 remain uninfected [5]. Given that it remains unclear how such a large proportion of HEU children resists infection, this topic warrants further study. 

These findings also highlight the need to understand the mechanisms of natural immunity to HIV-1 infection in the mother-newborn pair. We previously reported dysfunctional innate immune responses to Toll-like receptor (TLR) stimulation in HIV-1-infected mothers and newborns [6]. Moreover, activation of TLR7/TLR8 restores defective cytokine secretion by myeloid dendritic cells, but not by plasmacytoid dendritic cells, in both the mother and the cord blood (CB). The innate immune response has also been correlated with protection from infection during mother-to-child exposure [7], and this response has been proposed to be mediated by extracellular factors, mucosal or systemic IgA [8], α-defensins [9] and CCL3 [10]. 

To prevent HIV-1 viremia from reaching a certain threshold, intracellular antiviral restricting factors act at distinct phases of the viral cycle; this phenomenon has not yet been well studied in the mother-newborn pair. Importantly, the expression of intrinsic resistance factors is increased by type I interferon (IFN), which acts during both the early and the late stages of HIV infection [11]. Certain antiviral factors can also block the viral replication cycle before or during the process of reverse transcription, such as apolipoprotein B mRNA-editing enzyme catalytic polypeptide-like 3G (APOBEC3G) and tripartite motif 5α (TRIM-5α). APOBEC3G (A3G) inhibits HIV replication by inducing G-to-A hypermutation of the viral HIV-1 genome during reverse transcription [12]. As a counter-measure, the HIV-1 virion infectivity factor (vif) induces the degradation of A3G and A3F, thereby preventing these proteins from entering the budding virus and inducing proteasomal degradation [13,14]. 

Many TRIM proteins are induced by type I and type II IFNs, and several TRIM proteins are known to be required for the restriction of infection by lentiviruses [15]. High TRIM-5α expression induces structural disorder in the retroviral capsid, leading to the interruption of the natural “uncoating” process in a species-specific manner [16], and high expression of TRIM-5α has been associated with reduced susceptibility to HIV-1 infection [17]. TRIM22 blocks the intracellular trafficking of the viral structural protein Gag to the surface of the cell via an IFN-β-dependent mechanism [18]. In addition, tetherin (CD317) (Bst2/CD317/HM1.24) functions as an IFN-induced antiviral host protein that inhibits the release of many enveloped viruses by tethering virions to the cell surface; however, the effects of tetherin can be countered by the HIV viral protein U (Vpu) [19]. 

Stimulator of IFN genes (STING) is a critical signaling molecule involved in the innate response to cytosolic nucleic acid ligands. STING is also thought to sense membrane fusion events associated with viral entry in a manner that is independent of nucleic acid sensing [20,21]. Moreover, myxovirus resistance protein A (MxA), which is specifically induced by IFN-α, inhibits the viral life cycle at three distinct steps: nucleocapsid transport to the nucleus, transcription of viral gene products [22] and viral assembly (including HIV assembly) [23,24]. All of these antiviral factors are of interest for evaluation in mother-child HIV infection, although A3G is the only factor that has been examined to date [25]. Perinatally HIV-infected children are classified as progressors or long-term non-progressors according to criteria based on HIV viral load (VL) and CD4+ T cell counts over time. In contrast, no correlation has been observed between disease progression and A3G/A3F expression or hypermutation levels [25]. However, a genetic variant of the A3G genotype, H186 in GG, was significantly associated with more rapid declines in CD4+ T cell counts over time in ART-naïve HIV-infected Thai and Cambodian children with moderate immunodeficiency [26]. Recently, the A3G-H186R and F119F variants were also shown to be associated with altered HIV-1-related disease progression and central nervous system impairment in a cohort of 1,049 HIV-infected children [27]. 

The goal of this study was to characterize the expression patterns of antiviral restricting factors in different maternal compartments, such as peripheral blood mononuclear cells (PBMCs), decidua, placental chorionic villi and colostrum, and in fetal compartments, such as mononuclear CB cells. It remains unknown whether ART exposure during pregnancy alters the expression patterns of antiviral restricting factors in HIV-infected mothers and their newborns.

Altered mRNA gene expression and protein expression profiles were detected in both maternal and fetal placental tissues. These findings highlight the upregulation of antiviral factors in infected mother-newborn pairs, providing evidence of *in utero* induction of the innate immune response. 

## Materials and Methods

### Ethics

The research involving human participants that was reported in this study was approved by the São Paulo University Institutional Use Committee. The work was conducted in accordance with the Declaration of Helsinki. All mothers provided written consent on the behalf of the newborn participants. Ethics Committee has approved the consent procedure.

### Study population

This study enrolled 23 HIV-infected mothers from the obstetrics outpatient clinic at the Hospital das Clínicas, Faculdade de Medicina da USP (HC-FMUSP), and 21 HIV-1-uninfected (UN) mothers from the São Paulo University Maternity Hospital. The median ages were 26 years (range, 18-36) for the HIV-infected mothers and 31 years (range, 23-36) for the UN mothers. All HIV-1-infected mothers were undergoing ART, and they had received prophylactic intravenous zidovudine 3-5 h prior to cesarean section. Pre-term delivery was not an exclusion criterion. At the time of delivery, peripheral blood was collected from the mother and umbilical cord. All mothers had negative serology for syphilis, hepatitis B and C viruses, toxoplasmosis, cytomegalovirus, syphilis, rubella and herpes simplex viruses. The HIV-seronegative status of the UN mothers was confirmed, and they also gave birth by cesarean section. Colostrum was collected within 24 h of delivery and centrifuged. The cellular pellet was stored in RNAlater (Sigma, St Louis, MO, USA), at -20°C, and the supernatant was stored at -70°C.

### Isolation of mononuclear cells (MNCs) and placental tissues

Up to 4 h after delivery, MNCs from heparinized maternal venous blood and CB were isolated by Ficoll-Hypaque gradient centrifugation (GE Healthcare Bio-Sciences AB, Uppsala, Sweden) and stored in RNAlater. Serum samples from maternal venous blood and CB were also collected and stored at -70°C. The placental cotyledon, measuring 1 cm^2^, was cut from a random central area; all sections included maternal and fetal surfaces, which were separated macroscopically and stored in RNAlater at -20°C. After the tissue was homogenized with a TissueRuptor (Qiagen), the supernatants were used for RNA extraction.

### Real-time PCR

Total RNA was extracted from MNCs, placental tissues (maternal and fetal surfaces) and colostrum cells using an RNeasy Plus Mini Kit (Qiagen, Valencia, CA, USA), and reverse transcription was performed with a Sensiscript Reverse Transcriptase Kit (Qiagen). The primers used in the real-time PCR assay are detailed in Table 1. 

**Table 1:** Oligonucleotide primers used in the study for PCR amplification.

Primer	Sequence
APOBEC3F	5’- TGGAAGTTGTAAAGCACCACTCA - 3’ forward
	5’- AGCACCTTTCTGCATGACAATG - 3’ reverse
APOBEC3G	5’ - GGCTCCACA TAAACACGGTTTC - 3’ forward
	5’ - AAGGGAATCACGTCCAGGAA - 3’ reverse
TRIM-5α	5’- CTGGCATCCTGGGCTCTCAAAGT - 3’ forward
	5’- CATACCCCCAGGATCCAAGCAGTT - 3’ reverse
TRIM-22	5’- GGTTGAGGGGA TCGTCAGTA - 3’ forward
	5’- TTGGAAACAGATTTTGGCTTC - 3’ reverse
α1-defensin	5’ - GCAAGAGCTGATGAGGTTGC - 3’ forward
	5’ - GTTCCATA GCGACGTTCTTCC - 3’ reverse
MxA	5’- AAGCTGATCCGCCTCCACTT - 3’ forward
	5’- TGCAATGCACCCCTGTATACC - 3’ reverse
IFN-β	5’- GGCTGG CCCTGTGATATTTCTGTG - 3’ forward
	5’- ACCTGGCTCTCCTCCTCC CTTCCT - 3’ reverse
Tetherin	5’- CTGGGGATAGGAATTCTGGTGCTC - 3’ forward
	5’- CTCGCTGTTGGCCTTGATGGTGAA - 3’ reverse
STING	5’- ATATCTGCGGCTGATCCTGC - 3’ forward
	5’ - GGTCTGCTGGGGCAGTTTAT - 3’ reverse
GAPDH	5’ - GAAGGTGAAGGTCGGAGT - 3’ forward
	5’ - GAAGATGGTGATGGGATTTC - 3’ reverse

* Primers were synthesized at Life Technologies Custom Primers. GAPDH= glyceraldehyde-3-phosphate dehydrogenase

GAPDH mRNA levels in all samples were used to normalize the mRNA content. PCR was performed in an Applied Biosystems 7300 system using specific primers and SYBR Green (Applied Biosystems, Carlsbad, CA, USA) fluorescence detection reagents. The cycling protocol consisted of 10 min at 95°C, followed by 40 cycles of 15 s at 95°C and 60 s at 60°C. The amplification results were visualized and analyzed using Sequence Detection System (SDS) software (Applied Biosystems). Normalized expression was calculated as previously described by Livak [28].

### Western blotting

Placental tissues were lysed in RIPA and extraction buffers (Thermo Scientific, Waltham, MA, USA). The samples were then subjected to 4-12% sodium dodecyl sulfate polyacrylamide gel electrophoresis, followed by transfer to a nitrocellulose membrane (Amersham Biotech, Buckinghamshire, England). The antibodies used for protein detection were anti-human A3G, anti-hA3F and anti-hTRIM (Abcam, Cambridge, UK). The secondary antibodies were anti-rabbit IgG and anti-mouse IgG conjugated to horseradish peroxidase (Sigma-Aldrich). The immunoblots were visualized using the enhanced chemiluminescence substrate West Dura (Thermo Scientific). The nitrocellulose membranes were re-treated with Restore Western Blot Stripping Buffer (Thermo Scientific) and subjected to Western blotting using a monoclonal anti-β-actin antibody (Santa Cruz) to normalize the protein levels. The membranes were visualized using radiography, scanned using a Gel Doc EZ (Bio-Rad) and analyzed with Image Lab Software. The values were then expressed as intensity/density.

### Cytokine measurements

Serum and colostrum samples were assessed for CCL3 and CCL5 expression using an ELISA (RD Systems, Minneapolis, MN, USA) and for CXCL9, CXCL10, CCL2 and CXCL8 expression by cytometric bead array (Chemokine Kit; Becton Dickinson, San Jose, CA, USA) and flow cytometry (Fortessa; BD).

### Statistical analysis

The Mann-Whitney *U* test was used to compare variables between HIV-infected mothers and healthy controls, and the Wilcoxon signed-rank test was used for comparisons between paired samples, as maternal and CB samples. Spearman’s correlation was used to investigate the relationships between maternal and CB samples or placental tissues (decidua and chorionic villi). A value of p≤0.05 was considered significant.

## Results

### Participant characteristics

In total, 23 HIV-1-infected mothers (with their respective CB samples) and 21 healthy UN mothers were enrolled in this study (Table 2). All HIV-1-infected mothers were undergoing ART and had undetectable VLs (<50 copies/mL), with the exception of one mother (VL=1,911 copies/mL). The time of HIV-1 diagnosis ranged between 0 and 22 years, reflecting the mothers (2/23) who were diagnosed in the first trimester of pregnancy and those who were diagnosed 22 years ago. In the cohort of HIV-infected mothers, 5/23 acquired HIV through vertical transmission. In addition, two children from HIV-1-infected mothers seroconverted by the age of 15 months.

**Table 2:** Demographic details of the mothers and newborn.

	Mothers infected with HIV-1	Uninfected mothers
	N= 23	N=21
Maternal age (y)	26.0 ± 6.6*	32.2 ± 3.5
CD4 mm^3^	541.0 ± 245.8	
CD4 mm^3^ nadir	424.0 ± 218.5	
CD8 mm^3^	816.6 ± 437.3	
Viral load (copies/mL)	<50 **	
Time of HIV diagnosis (y)	9.4 ± 7.6	
Frequency of previous abortion	8/20	5/15
	Newborn from Mothers infected with HIV-1	Newborn from Uninfected mothers
Gestational age (weeks)	37.6 ± 0.5	39.2 ± 0.4
Gender	12 F/8 M	7 F/8 M
Birth weight (g)	2828.0 ± 390.9	3362.0 ± 356.6

* Media ± S.D.; ** 22/23

### Maternal blood and CB antiviral factor expression

Considering that the innate antiviral immune response plays a crucial role in the early phases of life, we evaluated the mRNA expression levels of antiviral factors in HIV-1-infected maternal blood and CB. CB from infected mothers was referred to as HEU. For the analysis, we selected particular factors related to restriction after viral entry but prior to viral integration into the nucleus, replication and egress, including A3G, A3F, TRIM-5α, TRIM-22, α1-defensin, MxA, STING, IFN-β and tetherin. 

**Figure 1:**
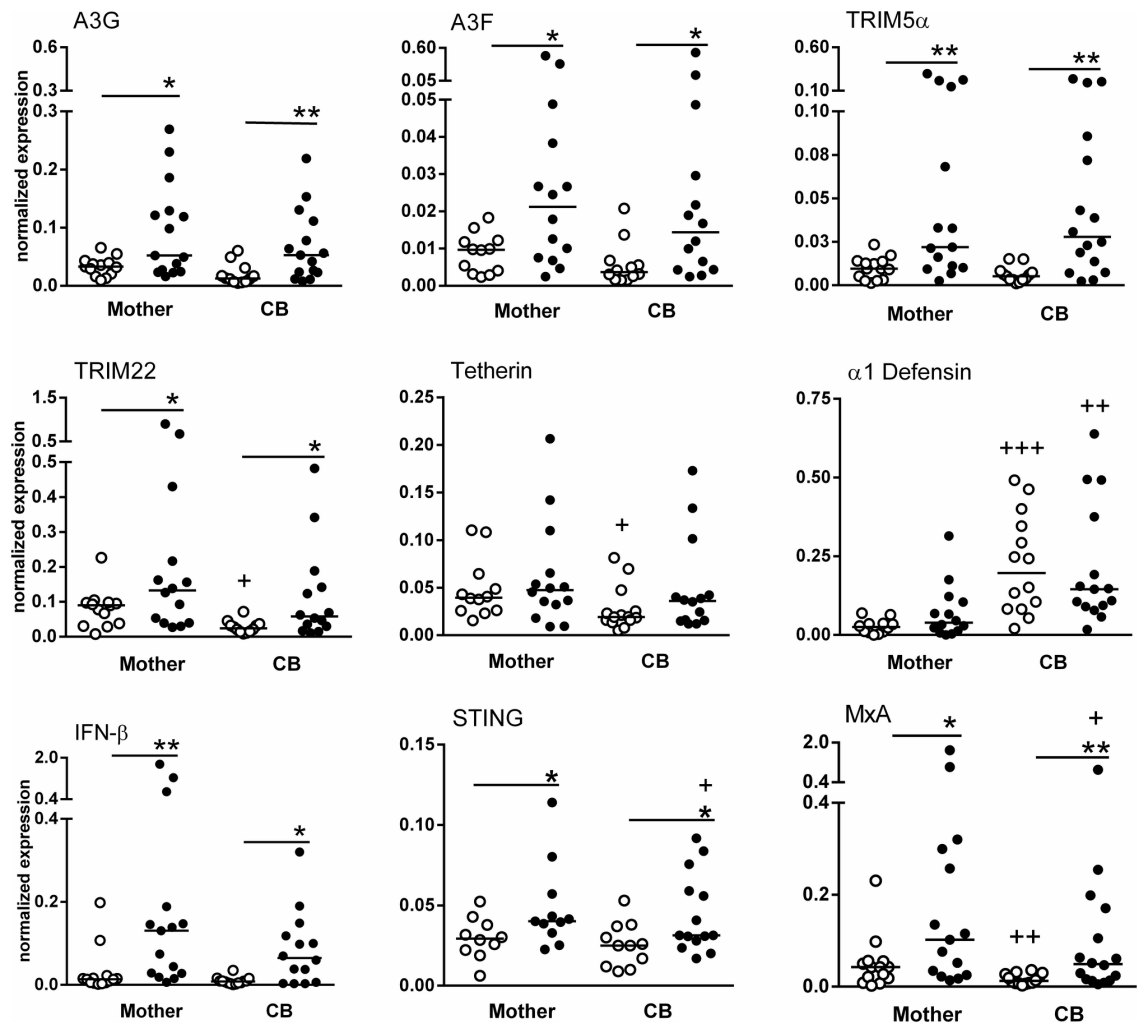
Upregulation of antiviral restricting factors in the MNCs of HIV-infected mothers and CB samples. The mRNA expression levels of antiviral factors, such as A3G, A3F, TRIM-5α, TRIM22, tetherin, α1-defensin, IFN-β, STING and MxA, in MNCs from HIV-infected mothers (closed symbols) (n=15) and corresponding CB (n=16) and in MNCs from UN mothers (open symbols) (n=13-14) and corresponding CB (n=11-13) were evaluated by real-time PCR. The data represent median values. *p≤0.05, **p≤0.01 compared with the control group; +p≤0.05, ++p≤0.01, +++p≤0.001 compared with the mother group.

Figure 1 shows that the mRNA expression levels of most antiviral factors were significantly increased in MNCs from HIV-1-infected mothers compared with MNCs from UN mothers (A3G, 0.09±0.08 vs 0.03±0.01, p=0.047; A3F, 0.07±0.14 vs 0.009±0.005, p=0.025; TRIM-5α, 0.07±0.09 vs 0.009±0.006, p=0.009; TRIM22, 0.22±0.26 vs 0.08±0.05, p=0.152; MxA, 0.28±0.47 vs 0.05±0.06, p=0.038; STING, 0.05±0.03 vs 0.03±0.01, p=0.044; and IFN-β, 0.31±0.51 vs 0.03±0.05, p=0.001; normalized expression, mean ± SD). However, the expression levels of tetherin and α-defensin were similar between the two groups. Of note, A3G, A3F, TRIM-5α, TRIM-22, IFN-β, STING and MxA expression levels were increased in CB from HIV-1-infected mothers as compared to CB from UN mothers, and these levels were similar to those observed in the maternal blood, with the exception of MxA and STING levels, which were decreased. The only factor expressed at a higher level in CB than in maternal blood was α-defensin, whose expression was increased 8.21-fold in the UN group and 2.96-fold in the HEU group (Figure 1). 

The samples from infected mothers that had vertically acquired HIV-1 infection who were included in the mRNA analysis (n=4) did not differ from the samples from other HEU individuals, as shown in Table S1.

 Next, we examined whether high antiviral factor expression levels in HIV-infected mothers were related to levels in the CB (Figure S1). Positive correlations between the expression levels of A3G (r^2^=0.604, p=0.02), A3F (r^2^=0.71, p=0.01), TRIM-5α (r^2^=0.72, p=0.002), MxA (r^2^=0.61, p=0.014) and IFN-β (r^2^=0.74, p=0.002) in HIV-infected mothers and their respective expression levels in HEU CB were detected. In contrast, only MxA expression was positively correlated between UN mothers and CB (r^2^=0.62, p=0.017).

### Placental antiviral factor expression

**Figure 2:**
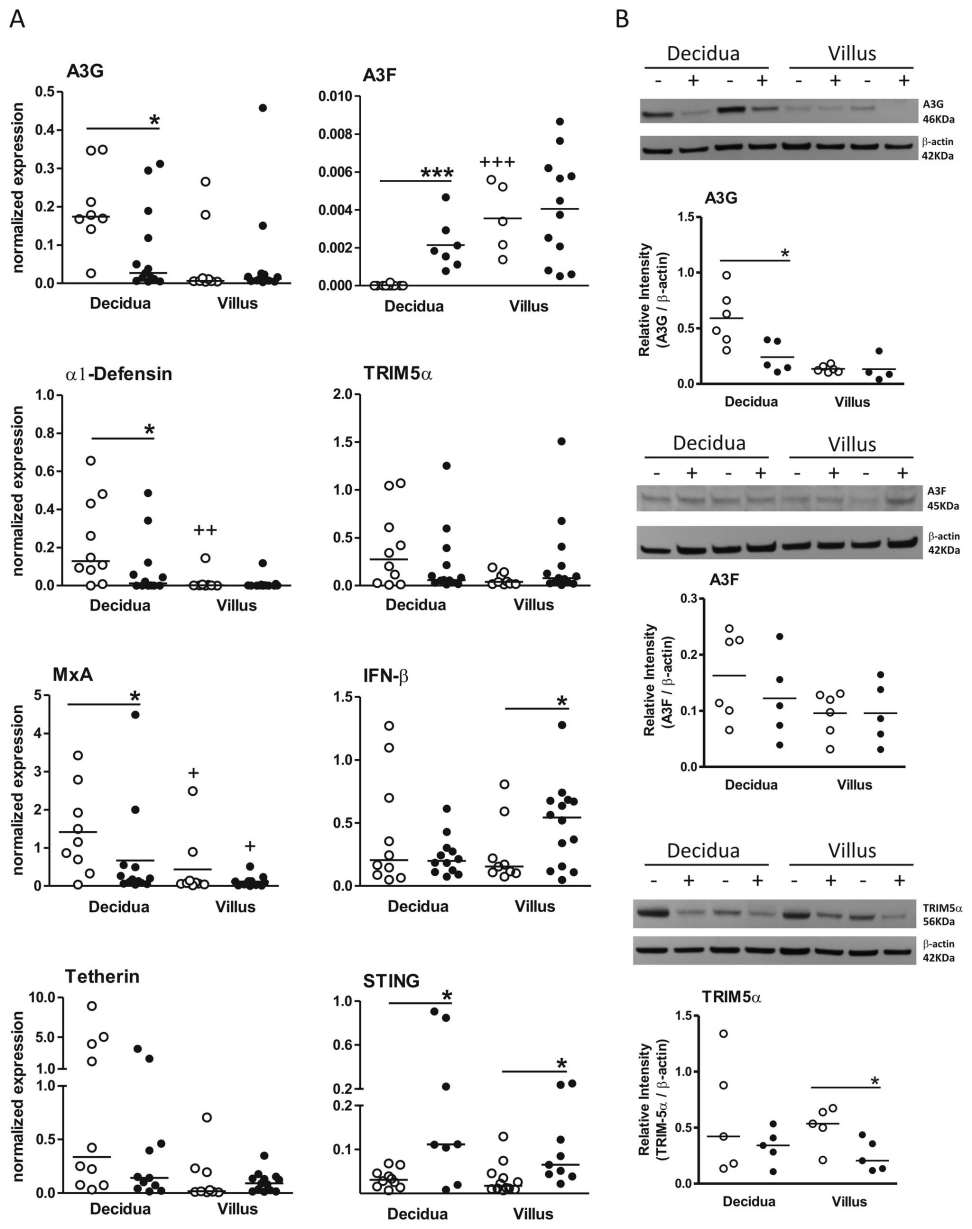
Differential expression of antiviral factors in the placental tissues of HIV-infected mothers and CB samples. A) Decidua (n=10-13) and placental chorionic villi (n=9-13) from HIV-infected mothers (closed symbols) and UN mothers (open symbols) were assessed for A3G, A3F, TRIM-5α, α1-defensin, IFN-β, tetherin and STING expression using real-time PCR. The data represent median values. B) Immunoblot analysis of A3G, A3F and TRIM5α protein in decidua (n=5-6) and villi (n=4-6) from infected mothers (+, closed symbols) and UN mothers (-, open symbols). The data represent median values.*p≤0.05 compared with the control group; ++p≤0.01 compared with the mother group.

We next evaluated the mRNA expression levels of antiviral factors in two types of placental tissue: the decidua (maternal face) and the chorionic villi (fetal face). A distinct pattern of antiviral factor mRNA expression was detected in the placental tissues of HIV-1-infected mothers compared with the expression observed in MNCs. Figure 2A shows a significant decrease in A3G, α1-defensin and MxA mRNA expression levels in the decidua of HIV-1-infected mothers compared with the decidua of the UN group (A3G, 0.08±0.11 vs 0.20±0.11, p=0.027; α1-defensin, 0.09±0.16 vs 0.23±0.22, p=0.044; and MxA, 0.67±1.26 vs 1.41±1.13, p=0.045). Interestingly, A3F was not detectable in the decidua of the UN group and was barely expressed in the decidua of HIV-1-infected mothers. Moreover, IFN-βexpression was increased in the fetal tissue associated with HIV-infected mothers. Only STING mRNA expression was significantly increased in both types of placental tissues obtained from HIV-infected mothers compared with UN tissues (decidua, 0.03±0.04 vs 0.29±0.36, p=0.033; villi, 0.03±0.02 vs 0.10±0.08, p=0.027). 

Next, we analyzed the protein expression levels of the representative antiviral factors A3G, A3F and TRIM-5α in placental tissues. Due to insufficient material for protein expression assessment, immunoblot analysis was performed on 5-6 decidua and 4-6 villi from HIV-infected mothers. Decreased A3G expression was observed in the decidua of the HIV-infected mothers, whereas decreased TRIM-5α protein expression was detected in their chorionic villi compared with expression in UN placentas (Figure 2B). In contrast, no differences in A3F expression were detected, and a similar pattern was observed in A3G transcript and protein levels in the decidua of infected mothers. 

### Colostrums Antiviral factor expression

**Figure 3:**
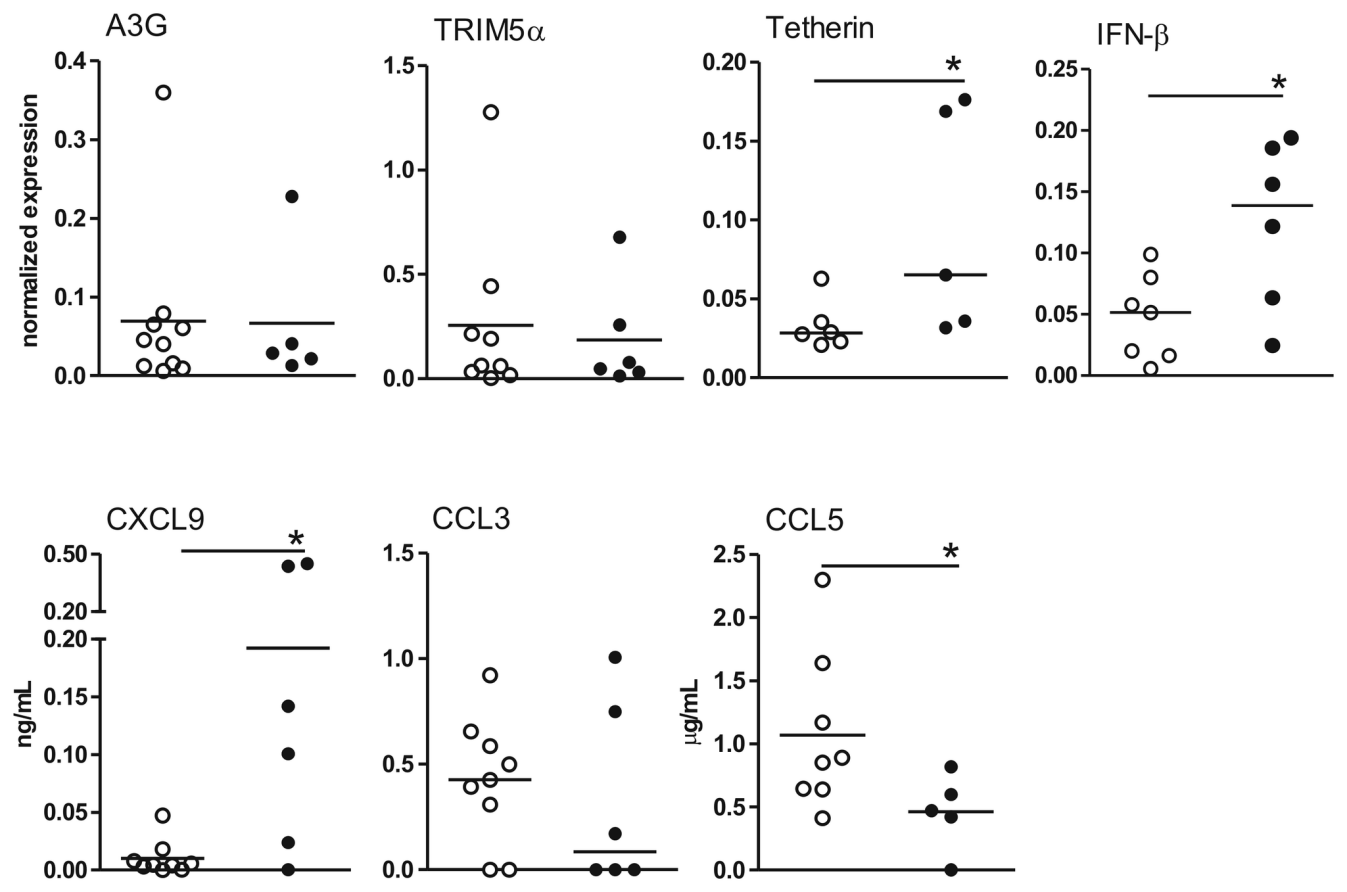
Extracellular and intracellular antiviral factors in colostrum from HIV-infected mothers and UN mothers. Colostrum samples were obtained up to 24 h after delivery from HIV-infected mothers (n=5-6) and UN mothers (n=9-10). The cells were evaluated by real-time PCR for A3G, TRIM-5α, tetherin and IFN-β mRNA expression or for CXCL9, CCL3 and CCL5 expression by flow cytometry. The data represent median values. *p≤0.05 compared with the control group.

 We collected colostrum up to 24 h postpartum and evaluated antiviral mRNA expression in the cell pellet and chemokine expression in the supernatant. Due to the recommendation to not breastfeed if HIV infected, it was difficult to obtain colostrum samples, so the analyses were performed on only 5-6 samples. Despite the low sample number, we observed that A3G and TRIM-5α mRNA expression levels in colostrum cells were similar between the groups of mothers, whereas tetherin and IFN-β mRNA levels were increased in HIV-infected mothers (Figure 3). In parallel, CXCL9 levels in colostrum from HIV-1-infected mothers were significantly increased, whereas CCL5 levels were decreased (Figure 3). Other chemokines, such as CXCL10/IP-10, CCL2/MCP-1, CXCL8/IL-8 and CCL3/MIP-1α, were detected at similar levels between the groups of mothers (data not shown). However, these results need to be confirmed in a larger sample.

### Chemokines Serum levels

**Figure 4:**
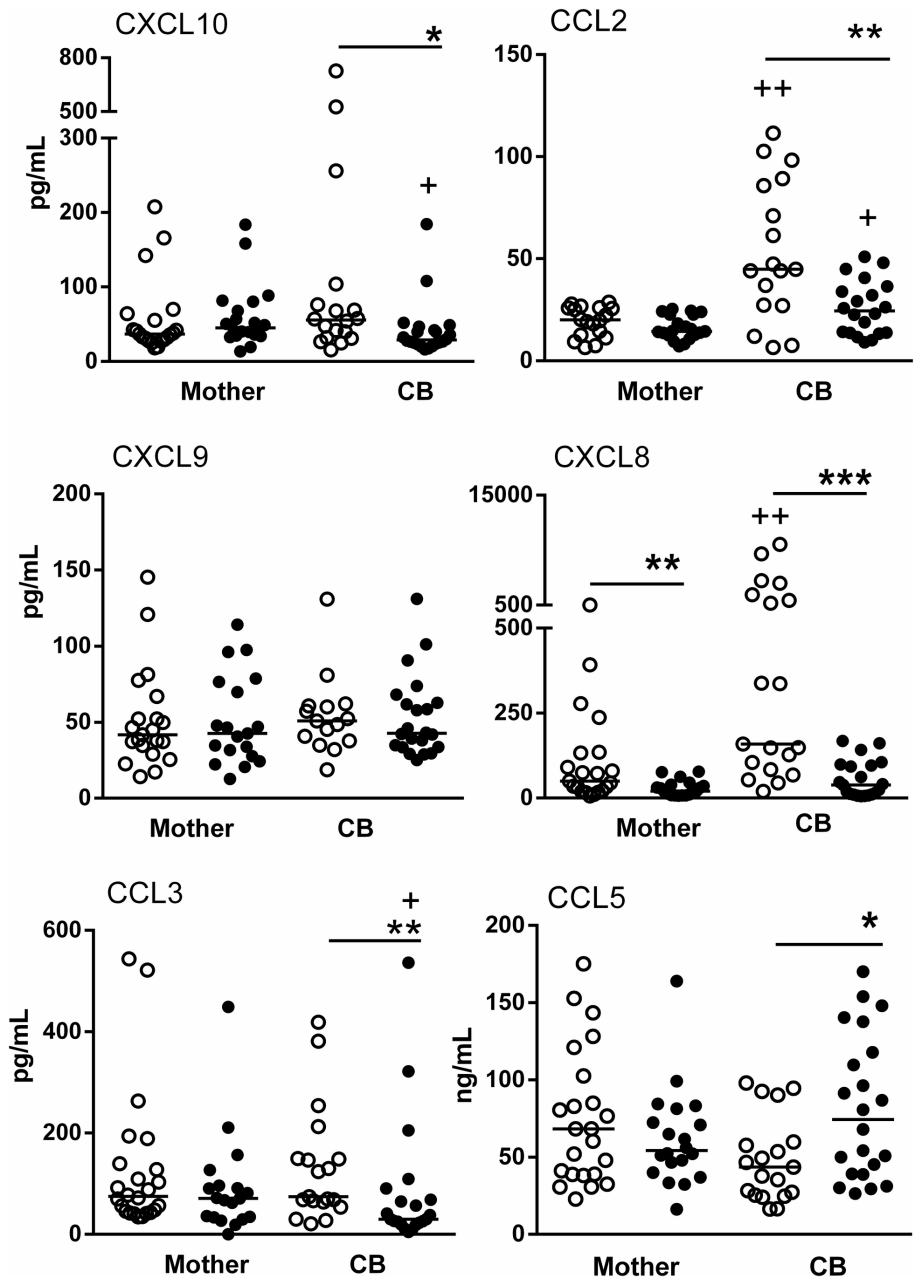
Altered chemokine levels in CB sera from HIV-infected mothers. Sera from HIV-infected mothers (closed symbols) and UN mothers (open symbols) (n=20-23) and their respective CB samples (n=20-23) were assessed for CXCL10, CCL2, CXCL9 and CXCL8 expression using a cytometric bead array, and CCL5 and CCL3 levels were determined by ELISA. The data represent median values. *p≤0.05, **p≤0.01, ***p<0.001 compared with the control group; +p≤0.05, ++p≤0.01 compared with the mother group.

We next measured the serum levels of chemokines in mothers and CB samples, including the β-chemokines CCL5 and CCL3. Figure 4 shows that HIV-infected mothers secreted similar chemokine levels as UN mothers, with the exception of decreased CXCL8 serum levels. Additionally, the serum levels of CXCL10, CCL2, CCL3 and CXCL8 were decreased in HEU CB. Curiously, CCL2 was detected at higher levels in CB sera from both groups compared with the levels in maternal blood. 

Of note, CCL5 levels were increased in CB from infected mothers compared with CB from UN mothers. We also evaluated CCL5 levels according the maternal nadir CD4+ T cell count, using CD4 counts of <500 cells/μL or >500 cells/µL because the latter number can serve as an indicator of increased or maintained CD4+ T cell levels (Figure S2). We observed that HIV-infected mothers with CD4+ T cell counts >500 cells/µL demonstrated significantly greater CCL5 serum levels (96±40.5 pg/mL) than mothers with counts <500 cells/μL (52±11.9 pg/mL). Additionally, there were no associations between CD4+ T cell counts and the other chemokines analyzed.

## Discussion

In this study, we explored the expression of antiviral restricting factors as an indicator of innate antiviral immunity in the HIV-infected mother-newborn pair. Although antiviral restricting factors were upregulated in the MNCs of infected vs. uninfected mother and CB, and altered patterns of transcription and protein expression were observed in placental tissues of HIV-1 infected vs. uninfected subjects. Moreover, colostrum cells exhibited particular patterns of endogenous antiviral factor expression that, together with extracellular factors, may indicate active natural antiviral immunity. 

Upregulation of A3G, A3F, TRIM-5α, TRIM22, IFN-β, STING and MxA mRNA expression was evident in the MNCs of HIV-infected mothers, and these mRNA gene expression levels were positively correlated with the levels in CB cells. These constitutive or inducible antiviral factors may be triggered by factors such as type I IFN, which was also upregulated at the transcript level. Notably, STING expression was increased in infected vs. uninfected CB cells as well. This transmembrane protein localizes to the endoplasmic reticulum and has been shown to play a critical role in retinoic acid-inducible (RIG-I) gene signaling, acting as a sensor of RNA virus infection [29]. In fact, many elements of the regulatory pathways governing both the RIG-I pathway and IFN gene expression are themselves regulated by IFN. In turn, IFN may coordinate the upregulation of antiviral factors, as revealed by the A3G, A3F, TRIM, tetherin and MxA mRNA expression levels in paired maternal blood and CB samples. Factors triggering antiviral factor expression may function *in utero*, although the gestational age at which this regulation may occur is unknown.

Despite the altered state of immunity of newborns, certain components of their innate immune systems are functional [30,31]. In addition, this study is the first to show that newborns can express endogenous antiviral factors that act at early stages of the viral cycle (such as A3G, A3F and TRIM5α), in the restriction of the late viral stage (such as TRIM22) or in both the early and the late viral stages (such as IFN-β together with MxA and STING). The upregulation of antiviral factors may indicate an important threshold for HIV protection that prevents intracellular HIV replication. However, although certain antiviral factors have been correlated with protection against HIV infection, others play the converse role. A genetic variant of *APOBEC3G*, genotype GG, was significantly associated with a more rapid decline in the CD4+ T cell count in ART-naïve HIV-infected Thai and Cambodian children with moderate immunodeficiency [26]. In contrast, the overexpression of human TRIM5α results in modest restriction of HIV-1 because this inhibition is insufficient to block the productive infection of human cells [32]. Moreover, HEU individuals and healthy controls exhibit similar mRNA expression levels for TRIM5α, A3G and tetherin in CD4+ T cells [33]. 

The exposure of HEU individuals to HIV has been shown to trigger A3G expression in PBMCs in the absence of infection, and cessation of HIV exposure is associated with decreased A3G expression [34,35]. Our cohort of HIV-1-infected mothers had undetectable VLs due to control by ART; however, exposure to HIV-1 could still occur. These findings suggest that an active innate immune response in newborns triggers the upregulation of antiviral factors, possibly due to the influence of maternal factors that induce an antiviral response.

Moreover, extracellular antiviral factors are crucial for the antiviral response, including the β-chemokines CCL3, CCL4 and CCL5. We observed increased CCL5 serum levels and decreased CCL3 levels in CB from infected mothers compared with CB from UN mothers. In particular, CCL5 levels were 900-fold greater than CCL3 levels in CB sera from HIV-infected mothers. CCL5 appears to be an immune correlate of protection because infants born to HIV-infected mothers who remain uninfected show higher levels of RANTES (regulated on activation, normal T cell expressed and secreted) than do infants with perinatal HIV-1 infection [35]. Despite the high levels of CCL5 in CB, we detected impaired CCL2, CXCL8 and CCL3 secretion compared with that observed in UN CB. It is possible that this altered production of chemokines, such as CXCL8, is associated with ART prophylaxis. In fact, it has been shown that ART prophylaxis is associated with significant anemia and neutropenia in HIV-uninfected infants during the first 3 months of life [36] and in women and newborns [37]. 

The profile of antiviral factor expression in the placental tissues of HIV-infected mothers differed between the decidua and the chorionic villi. Cells from the decidua showed decreased A3G and α-defensin mRNA expression levels, whereas cells from the chorionic villi revealed increased IFN-β levels. Interestingly, both placental faces showed increased expression of STING, a factor that is considered to be a major regulator of type I IFN [21]. Both placental and fetal membranes possess natural antiviral properties, as evidenced by their capacity to produce type I IFNs and antiviral peptides [38]. Additionally, trophoblast cells isolated during the first trimester constitutively express antiviral factors, such as 2’,5’-oligoadenylate synthetase, MxA, A3G, IFN-β and human β-defensin 1 [39]. 

Herein, we showed that full-term decidual cells may express A3G, TRIM-5α, α1-defensin, IFN-β, tetherin and STING and that villi express IFN-β, tetherin and STING at higher levels. In fact, the decidua exhibited greater alterations in expression levels because these tissues are rich in immune cells. For example, decidual natural killer (dNK) cells constitute 20% of the total leukocyte population [40], and approximately 10% of the decidual leukocyte population consists of T cells [41], including the TCD8, γδT, natural killer T (NKT) and innate regulatory T subsets, all of which are crucial for fetal tolerance. Placentas from infected mothers exhibited decreased protein expression of A3G in the decidua and TRIM-5α in the villi, although TRIM-5α transcript levels were similar. However, it is not clear whether the decrease in A3G protein was due to *vif* degradation and whether the decrease in TRIM-5α was due to an unknown HIV-related counteracting factor, so further research is required to explore these possibilities. 

Cells from the colostrum of HIV-infected mothers showed increased tetherin and IFN-β mRNA expression levels. Antiviral factors such as tetherin, an IFN-induced protein, block the release of HIV-1 [19,42]. Nevertheless, depending on the cells in which it is expressed, tetherin can be correlated with disease progression. An increased tetherin level in CD4+ T cells in untreated HIV-1-infected patients has been reported as a marker of advanced HIV infection [33]. Moreover, in the current study, colostrum samples from HIV-infected mothers showed decreased CCL5 levels and increased CXCL9 levels, most likely indicating a protective mechanism. An increased CCL5 level in milk has been associated with a higher transmission risk [43]. In addition, CXCL9 in human milk, which is inducible by IFN-γ, is most likely derived from milk cells or mammary gland epithelial cells and contributes to the migration and activation of intestinal T lymphocytes that enhance mucosal immunity during the early neonatal period [44].

Taken together, the data presented here indicate that the mRNA gene and protein expression of natural antiviral factors in HIV-infected mother-newborn pairs are enhanced. Moreover, intracellular and extracellular factors that are constitutive or inducible by type I IFN could represent crucial mechanisms in the antiviral response of the pregnant mother and her fetus, whereas other immunological factors could be impaired by either ART or HIV exposure. These findings suggest that employing immune modulators that activate the innate immune response could constitute a strategy for triggering the stimulation of IFN genes and exert antiviral immunity.

## Supporting Information

Figure S1 **Correlation of A3G, TRIM-5α, MxA and IFN-β levels between HIV-infected mothers and their corresponding CB and of MxA levels between UN mothers and their corresponding CB.**
(TIF)

Figure S2 **CCL5 serum levels in HIV-infected mothers and their corresponding CB according the maternal nadir CD4+ T cell count, using counts of <500 cells/μL and >500 cells/µL.**
(TIF)

Table S1 **A similar profile of antiviral mRNA expression was observed in PBMCs between mothers who were HIV-1 infected by vertical transmission and HIV-1-infected mothers.**
(DOCX)
