# Supplementary figures and images for: Upregulation of Innate Antiviral Restricting Factor Expression in the Cord Blood and Decidual Tissue of HIV-Infected Mothers
Source: PLoS One. 2013 Dec 18;8(12):e84917. doi: 10.1371/journal.pone.0084917 (PMC3867518; doi:10.1371/journal.pone.0084917)

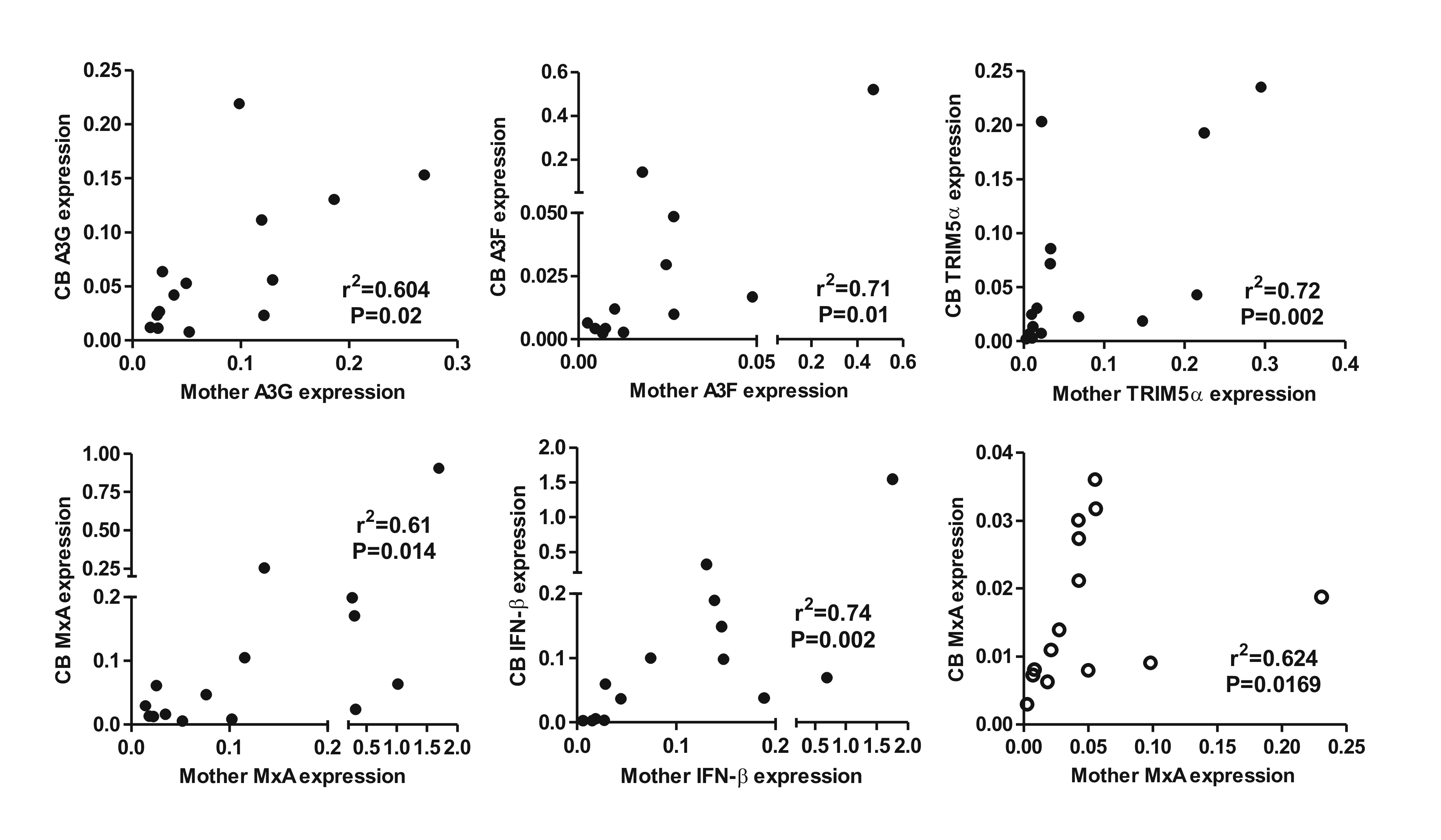

Supplement: Figure S1 — Correlation of A3G, TRIM-5α, MxA and IFN-β levels between HIV-infected mothers and their corresponding CB and of MxA levels between UN mothers and their corresponding CB. (TIF) [file pone.0084917.s001.tif]

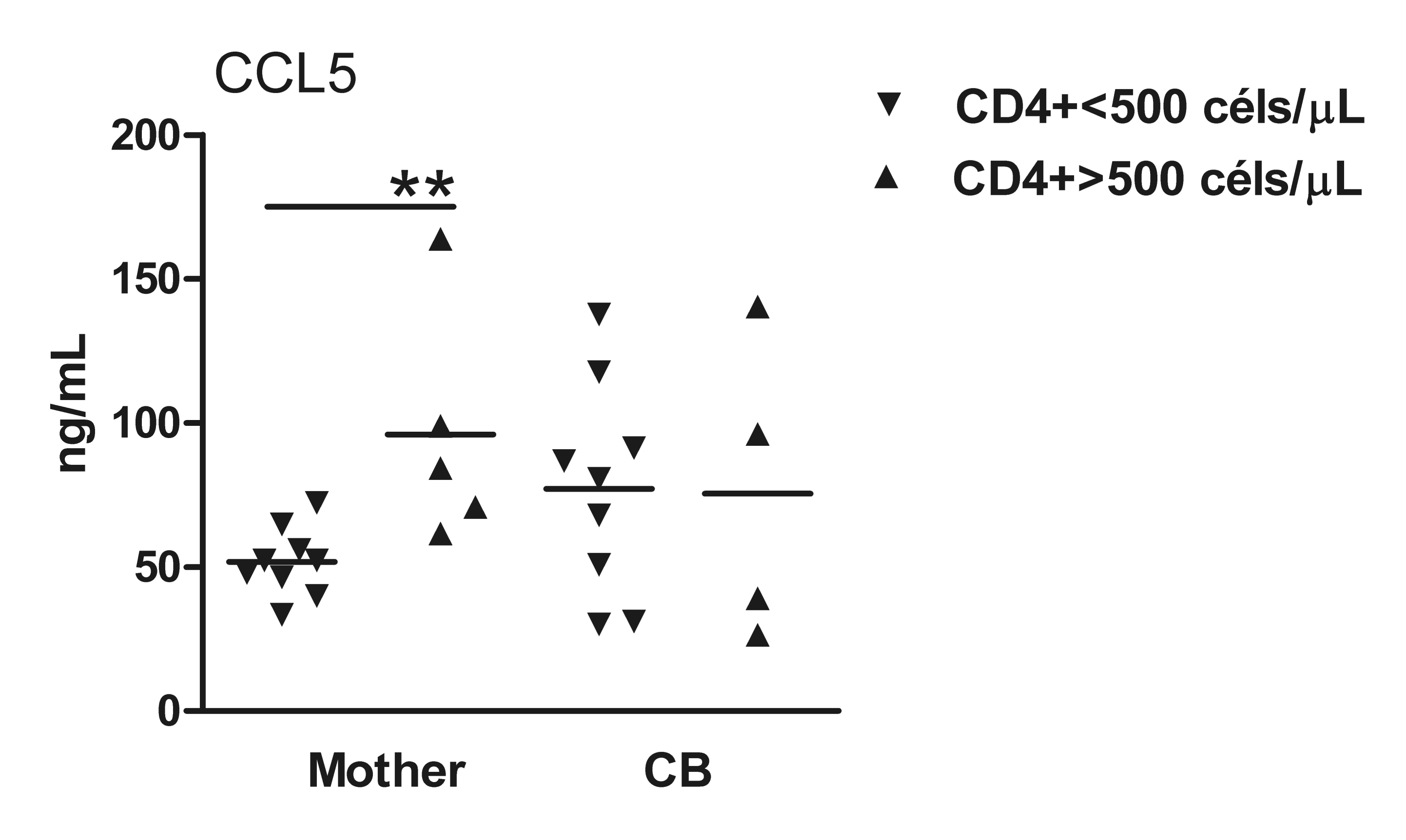

Supplement: Figure S2 — CCL5 serum levels in HIV-infected mothers and their corresponding CB according the maternal nadir CD4+ T cell count, using counts of <500 cells/μL and >500 cells/µL. (TIF) [file pone.0084917.s002.tif]
