# Supplementary material for: Upregulation of Innate Antiviral Restricting Factor Expression in the Cord Blood and Decidual Tissue of HIV-Infected Mothers
Source: PLoS One. 2013 Dec 18;8(12):e84917. doi: 10.1371/journal.pone.0084917 (PMC3867518; doi:10.1371/journal.pone.0084917)
Supplement: Table S1 — A similar profile of antiviral mRNA expression was observed in PBMCs between mothers who were HIV-1 infected by vertical transmission and HIV-1-infected mothers. (DOCX) [file pone.0084917.s003.docx]

**Table S1. A similar profile of antiviral mRNA expression was observed in PBMCs between mother HIV-1-infected by vertical transmission and HIV-1-infected mothers**

| **Antiviral factor**  **mRNA expression** | **Mother HIV-infected by vertical transmission**  **(n=4)** | **Mother HIV-infected**  **(n=11)** | ***P* value** |
| --- | --- | --- | --- |
| A3G | 0.11 ± 0.05 | 0.52 ± 0.05 | 0.13 |
| A3F | 0.05 ± 0.06 | 0.06 ± 0.16 | 0.30 |
| TRIM 5α | 0.09 ± 0.08 | 0.05 ± 0.08 | 0.20 |
| TRIM22 | 0.20 ± 0.20 | 0.08 ± 0.10 | 0.14 |
| Alpha-defensin | 0.28 ± 0.18 | 0.19 ± 0.19 | 0.32 |
| MxA | 0.11 ± 0.10 | 0.13 ± 0.25 | 0.24 |
| IFN-β | 0.16 ± 0.11 | 0.06 ± 0.06 | 0.08 |
| Tetherin | 0.10 ± 0.07 | 0.05 ± 0.05 | 0.08 |
| STING | 0.04 ± 0.02 | 0.04 ± 0.02 | 0.57 |

*Mean normalized expression ± S.D.
